# Synchronous and asynchronous tele-exercise during the coronavirus disease 2019 pandemic: Comparisons of implementation and training load in individuals with spinal cord injury

**DOI:** 10.1177/1357633X20982732

**Published:** 2021-01-18

**Authors:** Rodrigo Rodrigues Gomes Costa, Jefferson R Dorneles, João HCL Veloso, Carlos WP Gonçalves, Frederico Ribeiro Neto

**Affiliations:** SARAH Network of Rehabilitation Hospitals, Brazil

**Keywords:** Health plan implementations, pandemics, rehabilitation, resistance training, telehealth, workload, COVID-19, pandemic

## Abstract

**Introduction:**

Tele-exercise could represent an alternative for remote care in individuals with spinal cord injury at this time of the pandemic of coronavirus disease 2019. However, the differences regarding the training loads and implementation between synchronous and asynchronous types are not yet known. The purpose of this study was to compare the implementation and training load between synchronous and asynchronous tele-exercise programs in individuals with spinal cord injury.

**Methods:**

Forty individuals with spinal cord injury were recruited and stratified into tetraplegia and paraplegia groups. All subjects performed 3 weeks of both the synchronous and asynchronous tele-exercise programs, after two weeks of familiarization with the exercises, remote connection tools and methods to record information. The primary outcomes were training load (average daily workload and average and total weekly training load) and implementation (adherence and successful exercise recording). Demographic characteristics were obtained from participants' electronic medical records.

**Results:**

Weekly mean workload, total workload, adherence and successful exercise recording presented significantly higher values in the synchronous compared to asynchronous tele-exercises. Average daily workload did not present significant differences between the tele-exercises.

**Discussion:**

The training load for each training session presented no differences between synchronous and asynchronous tele-exercises. Both adherence and successful data recording showed more favourable implementation values for synchronous training, thus allowing greater weekly training loads (total and average).

## Introduction

The pandemic of coronavirus disease 2019 (COVID-19) has induced world governments to adopt strict rules that limit individual freedom and impose social distancing (e.g. closing schools, mandatory quarantine, restricting entertainment) in order to prevent the collapse of care in national health systems.^1,2^ Although these measures are necessary during pandemics in order to prevent the spread of infection, many individuals worldwide are also consequently deprived of access to health-related interventions or monitoring.^1,3^

Individuals with spinal cord injury (SCI) are at a higher risk of contracting and developing morbidities due to COVID-19, arising from the difficulty of early diagnosis due to physiological alterations caused after SCI,^4^ greater vulnerability to respiratory infections^5,6^ and the need for a caregiver for daily activities.^7^ Therefore, planning for access to health and social isolation are important measures for individuals with SCI during the pandemic. On the other hand, physical inactivity can also cause harmful health effects. In addition, physical inactivity and strenuous exercise are related to depression of the immune system, while moderate exercise is associated with a reduced incidence of infections.^8–13^ In particular, for individuals with SCI to present the benefits of exercise to health promotion, aerobic exercise is recommended at least twice a week for 20 min, in addition to the practice of muscle strengthening exercises, twice a week, both with moderate to high intensity.^14^ Thus, creating physical activity prescription methods for individuals with SCI during the pandemic is important for maintaining the health benefits provided by exercise.

In order to provide alternative health-related interventions, recent innovations allow healthcare professionals to provide services remotely through communication technologies (for example, smartphone or video call via computers with Internet access), known as telehealth.^15,16^ With regard to individuals with SCI, telehealth has been shown to help in the treatment of pressure injuries^17^ and implementation of strategies to promote healthy behaviours.^18^ A subdivision of telehealth is tele-exercise, defined as interventions that offer physical training and are provided remotely.^16^ Lai et al.^16^ found high acceptability for an aerobic exercise program, which was attributed to its accessibility, convenience and interpersonal interaction with the professional.

**Figure 1.**
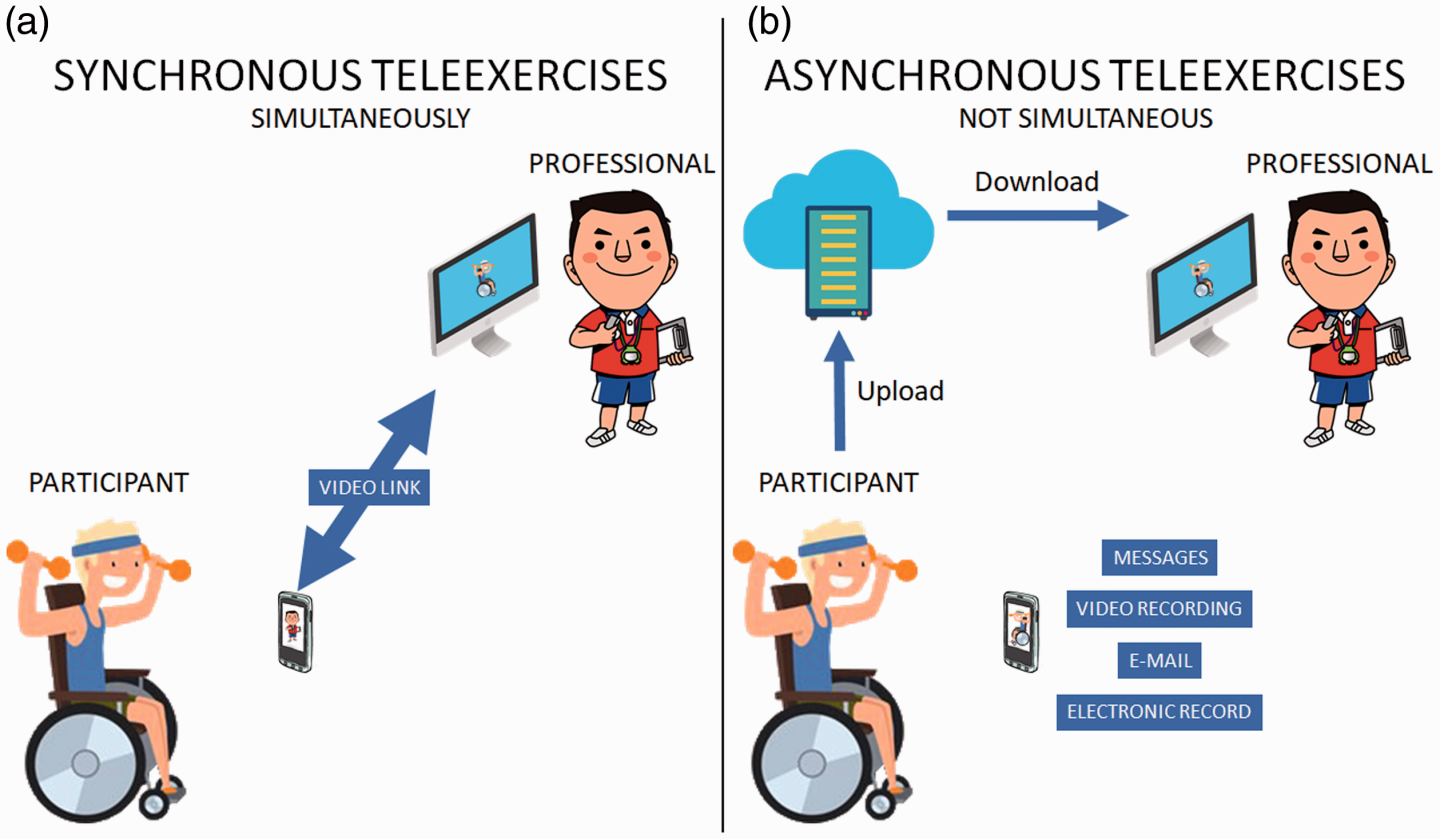
Differences between (a) synchronous and (b) asynchronous tele-exercises.

Tele-exercise, like telehealth, can be divided into two types: synchronous and asynchronous. The first is characterised by a real-time approach, in which the service takes place simultaneously between the patient and the professional by video conference or audio or text phone conversations (Figure 1(a)).^19^ The second is the asynchronous model that provides an alternative to traditional synchronous technologies, allowing communication without the need for real time (for example, email and other messaging systems) (Figure 1(b)).^19^ One of the ways to evaluate the applicability of remote interventions is through implementation, that is, the effectiveness of implementation through the variables adherence and successful data registration.^16^

For both synchronous and asynchronous tele-exercises, control of the training load can assist in its prescription, aiming to avoid very low intensities of physical activity which will not provide the expected health benefits, as well as high intensities of training which can lead to a risk of injury.^20–23^ One way of monitoring the training sessions to measure the intensity of the exercise and to avoid the risk of injury is to control the internal load that enables the evaluation of physiological and psychological stresses imposed on the practitioner.^24–26^ On the other hand, the external load is the amount of the workload regardless of the internal characteristics.^25,27^ One of the methods to monitor the internal load was proposed by Foster et al.,^28^ using the rating of perceived exertion (RPE) associated with training session time. RPE is characterised as the perception of how strenuous a physical task is.^29^

Tele-exercise could represent an alternative for remote care in individuals with SCI at this time of the COVID-19 pandemic, however, the differences regarding the training loads and implementation between synchronous and asynchronous types are not yet known. Recently, Bombardier et al. suggested studies with more objective indicators of physical activity frequency, intensity and duration.^30^ In addition, it is relevant to verify the differences between the types of tele-exercise with the aim of providing a greater basis in relation to the implementation of these new technologies and how they can be monitored through the analysis of the training load, avoiding situations of strenuous exercises in the context of the COVID-19 pandemic.

Thus, the objective of the present study was to compare the implementation and training load between synchronous and asynchronous tele-exercise in individuals with SCI. The hypothesis of the present study was that the outcomes related to the training load and implementation would be greater in synchronous training compared to asynchronous training.

## Methods

### Participants

Forty men and women with SCI were consecutively recruited from the rehabilitation programme of the Network Centre of Rehabilitation Hospitals. Data were collected in May and June 2020. The study was approved by the institutional Ethics Committee (protocol n. 4.268.841). All participants were outpatients and provided written informed consent.

Inclusion criteria were: (a) adult individuals (from 18 years old) of both sexes; (b) individuals who participated in a spinal cord rehabilitation programme in person prior to the COVID-19 pandemic; (c) diagnosis of at least 1 year of traumatic or non-traumatic (non-progressive) SCI with classification of the severity (i.e. completeness) of injury by the American Spinal Injury Association Impairment Scale (AIS)^31^ ranges to A or B (complete motor injury); (d) participants with Internet access with sufficient capacity to follow the video lessons; and (e) those who had participated in at least 2 weeks of tele-exercise practices in synchronous form. Participants were excluded if they had a history of metabolic disorders, cardiovascular, cardiac or orthopaedic surgery that would hamper performance in the tele-exercises. Participants were stratified into paraplegia and tetraplegia groups.^31^

The present study used the checklist Template for Intervention Description and Replication (TIDieR).

### Procedures

The participants performed synchronous and asynchronous tele-exercises for 6 weeks, three times a week, with the objective of training muscle strength. The synchronous exercises were performed in the first place to ensure the safety of the participants in the asynchronous tele-exercises. Synchronous tele-exercise were delivered using the free teleconference application (app) (Google Meets software) and asynchronous tele-exercise were delivered using the free message app (WhatsApp software). The groups of tele-exercises were private and the professional sent the link for each training session and controlled the access of the participants. The participants performed the synchronous tele-exercises at their own residence in the paraplegia or tetraplegia group according to their level of injury. The paraplegia group had one teacher and the tetraplegia group had another teacher. These teachers were physical educators with spinal cord injury expertise and these teachers were the same in all sessions.

The exercises involved the muscle groups of the anterior and posterior trunk (major pectoralis, latissimus dorsi, biceps brachii, triceps brachii and deltoids) using implements such as dumbbells, shin guards or the body mass itself. In both tele-exercises, three sets of 10–15 repetitions were performed according to the implements available at the participant's residence. The activities were part of the rehabilitation programme proposed by the Network Center of Rehabilitation Hospitals, during the COVID-19 pandemic.

**Figure 2.**
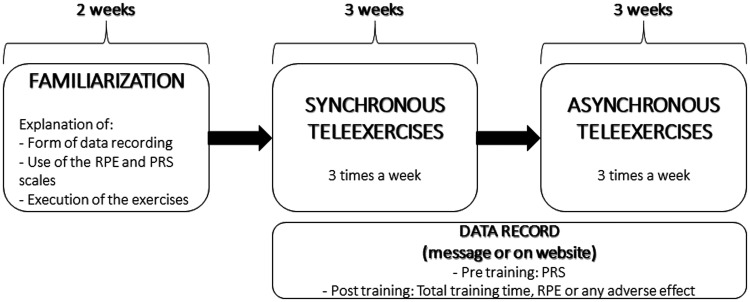
Flowchart of familiarization and training weeks. PRS: perceived recovery scale; RPE: rating of perceived exertion.

Initially, 2 weeks of familiarization and preparation were allowed, in which the participants were informed about how to recording of the data, in addition to performing synchronous training to learn the exercises and become familiar with the scales and the software related to the video call. Next, the participants underwent 3 weeks of synchronous tele-exercises followed by another 3 weeks of asynchronous tele-exercises. Synchronous tele-exercises took place in groups through video calls, accompanied by the physical education teacher. In asynchronous tele-exercises, patients performed the same exercises practised in the three synchronous weeks, individually, without the real-time presence of the teacher during the moment of execution. The recordings were made through a questionnaire link on the Internet provided by a message on the cell phone or by sending direct messages via the cell phone with the requested data (Figure 2).

In all tele-exercise sessions, participants were asked about the presence of a disabling injury or pain, defined as any condition that would prevent participation in the exercise programme.^32^ In addition, participants could send direct messages via the cell phone or communicate via the Internet link available in all tele-exercise sessions.

#### Quantification of training workload

The internal training workload was calculated for each training day using the method proposed by Foster et al.^28^ (multiplying total session time in minutes by training intensity). Training intensity was measured using the Borg scale of perceived exertion (Category-Ratio scale anchored at number 10; CR10)^29,33^ 30 min after the end of each session, as proposed by Foster et al.^28^ to calculate the internal training load. The internal training workload was expressed in arbitrary units (AU) in three ways: (a) average daily workload (average training workload performed during the week); (b) average weekly workload (average weekly training workload performed for the 3 weeks); and (c) total weekly training workload (sum of the workload of all the training sessions for the 3 weeks).

#### Perceived recovery scale (PRS)

Immediately before the training sessions, the participants performed an assessment using the PRS.^34^ Similar to the RPE, the scale aims to assess the perceived recovery of the participants before the training sessions, in order to adjust daily training intensities, thereby minimising risks of injury, overreaching or overtraining. The PRS is a numerical scale from 0–10, where zero corresponds to ‘very poorly recovered/extremely tired’ and 10 to ‘very well recovered/highly energetic’.^34^

#### Implementation

The implementation was evaluated by the variable adherence and successful registration of data.^16^

Adherence to the intervention was defined as the percentage of exercise sessions performed in relation to the total sessions proposed.^16^

Successful data recording was defined as the percentage of sessions that were monitored and recorded^16^ through completion of an Internet link or by sending direct messages via cell phone. A successful recording of the exercise required that all training session data were successfully saved, including RPE, PRS and total training time.

### Patient and public involvement

Patients and/or the public were not involved in the design, conduct, reporting or dissemination of the plans of this research.

### Outcomes

The primary outcomes were implementation (adherence and successful exercise recording) and training load (average daily workload, average weekly workload and total workload).

Demographic characteristics (age at injury, birth date, aetiology, neurological level of injury, sex and time since injury) were obtained from participants' electronic medical records.

### Statistical analysis

Sample size was calculated based on the Wilcoxon signed-rank test, considering an effect size of 0.50, α of 5%, and power (1–β) of 85%, demonstrating that 40 individuals were required to compare synchronous and asynchronous tele-exercises.

The Kolmogorov-Smirnov test was used to assess the data distribution. Descriptive data are presented as median and interquartile (25th and 75th percentiles) for all outcomes. The within-group analysis (synchronous vs asynchronous teleexercises) was conducted using the Wilcoxon test. The Mann-Whitney test compared the primary outcomes between paraplegia and tetraplegia groups. A chi-square test was used to compare the frequency proportions of SCI level and aetiology between paraplegia and tetraplegia.

The outlier labelling rule was used to detect outliers and discrepancies.^35^ Outlier values were calculated by the difference between the 25th and 75th percentiles multiplied by a factor (2.2). The result was then subtracted from the 25th percentile and added to the 75th percentile.

The IBM SPSS Statistics package (version 22.0; SPSS Inc, Armonk, New York, USA) and G*Power statistical power analysis software (version 3.1.9.2; Universität Kiel, Germany) were used. Statistical significance was set at 5% (*p* ≤ 0.05; two-tailed).

## Results

### Participant characteristics

**Figure 3.**
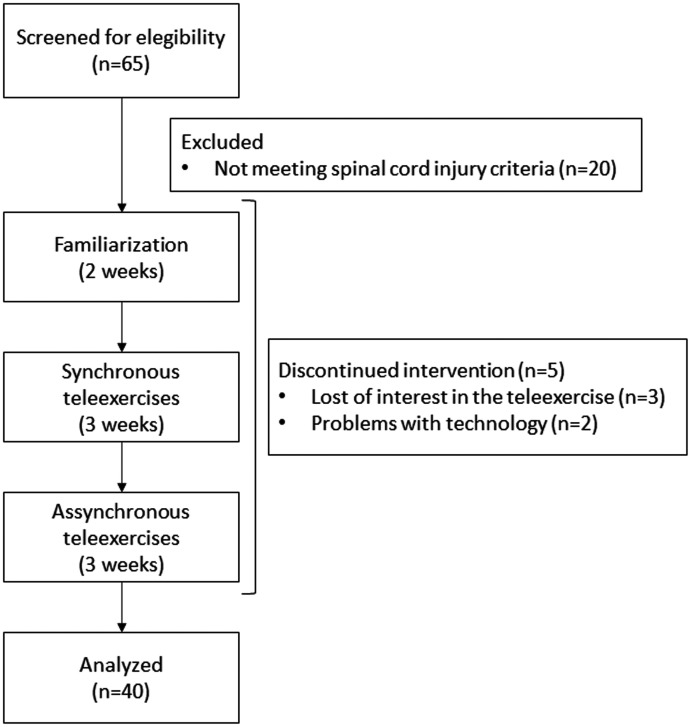
Flowchart diagram.

Of 65 participants who were screened, 14 were excluded upon eligibility criteria and 11 were discontinued from the intervention (flow chart displayed in Figure 3). There were no dropouts from this study, and no significant differences were found in age, time since injury and age at injury between paraplegia and tetraplegia groups. The sex and disability distribution did not present significant differences between groups (Table 1). No disabling pain or injuries were reported during synchronous and asynchronous tele-exercises.

**Table 1.** Participant demographic data presented as median (25th and 75th percentiles) for paraplegia and tetraplegia groups. Sex and disability are expressed in absolute values (frequency).

	Total	Paraplegia	Tetraplegia
*n*	40	20	20
Injury level	C5 to L1	T4 to L1	C5 to C8
Age (years)	36.0 (26.0–42.9)	36.0 (25.3–38.4)	36.6 (26.9–44.4)
Time since injury (months)	74.5 (32.4–167.7)	61.3 (24.3–167.7)	102.5 (46.3–169.5)
Age at injury (years)	22.5 (19.2–32.1)	22.5 (20.4–32.5)	22.0 (18.6–31.4)
Sex (*n*)			
Female	22 (55.0%)	7 (35.0%)	15 (75.0%)
Male	18 (45.0%)	13 (65.0%)	5 (25.0%)
Aetiology (*n*)			
Auto accident	23 (57.5%)	10 (50.0%)	13 (65.0%)
Bacterium	1 (2.5%)	1 (5.0%)	0 (0.0%)
Congenital	1 (2.5%)	0 (0.0%)	1 (5.0%)
Diving	3 (7.5%)	0 (0.0%)	3 (15.0%)
Falls	2 (5.0%)	1 (5.0%)	1 (5.0%)
Gunshot wound	8 (20.0%)	7 (35.0%)	1 (5.0%)
Motorcycle accident	1 (2.5%)	1 (5.0%)	0 (0.0%)
Tumour	1 (2.5%)	0 (0.0%)	1 (5.0%)

### Implementation

The adherence and successful exercise recording presented significantly higher values in the synchronous compared to asynchronous tele-exercises (66.7% vs 50.0% and 100.0% vs 71.4%, respectively). There were no significant differences between paraplegia and tetraplegia groups (Table 2).

**Table 2.** Comparisons of training and monitoring outcomes of synchronous and asynchronous tele-exercises for paraplegia and tetraplegia groups. The outcomes are presented as median (25th and 75th percentiles).

	Total	Paraplegia	Tetraplegia
Synchronous			
Perceived recovery scale	8.0 (7.4–8.8)	8.4^a^ (7.8–9.2)	7.8 (7.0–8.1)
Rating of perceived exertion	5.7 (4.8–6.7)	6.6^a^^,b^ (5.7–7.0)	4.9 (3.5–5.7)
Total time of training	25.3 (22.2–26.8)	26.7^a^ (26.0–28.1)	21.7 (20.0–23.3)
Training load (AU)			
*Daily average*	152.1 (108.3–175.8)	174.6^a^ (156.6–187.2)	108.6 (82.5–121.1)
*Weekly average*	246.9^b^ (151.1–335.0)	279.2^a^^,b^ (182.1–393.1)	189.2 (86.7–290.0)
*Total workload*	740.5^b^ (410.0–1005.0)	837.5^a^^,b^ (546.2–1179.3)	555.0^b^ (260.0–870.0)
Adherence	66.7%^b^ (38.9–77.8%)	52.8% (44.4–77.8%)	72.2% (33.3–83.3%)
Successful exercise recording	100.0%^b^ (100.0–100.0%)	100.0%^b^ (100.0–100.0%)	100.0% (75.0–100.0%)
Asynchronous			
Perceived recovery scale	8.0 (7.4–8.7)	7.0 (8.0–8.6)	8.0 (7.7–8.7)
Rating of perceived exertion	5.5 (4.9–6.0)	5.3 (5.7–6.0)	5.3 (4.9–6.0)
Total time of training	22.7 (20.0–27.6)	20.0 (26.3–33.3)	22.7 (19.0–24.8)
Training load (AU)			
*Daily average*	125.6 (105.6–147.0)	140.0 (116.5–170.0)	121.5 (92.6–135.4)
*Weekly average*	153.3 (0.0–270.0)	98.3 (0.0–333.8)	190.3 (30.0–247.0)
*Total workload*	397.0 (0.0–718.0)	295.0 (0.0 –1001.3)	511.0 (48.0–705.0)
Adherence	50.0% (5.6–77.8%)	38.9% (0.0–77.8%)	61.1% (16.7–77.8%)
Successful exercise recording	71.4% (33.3–100.0%)	71.4% (26.7–100.0%)	100.0% (57.1–100.0%)

^a^Significant difference compared to tetraplegia group (*p* ≤ 0.05); ^b^significant difference compared to asynchronous training (*p* ≤ 0.05).

### Training load

**Figure 4.**
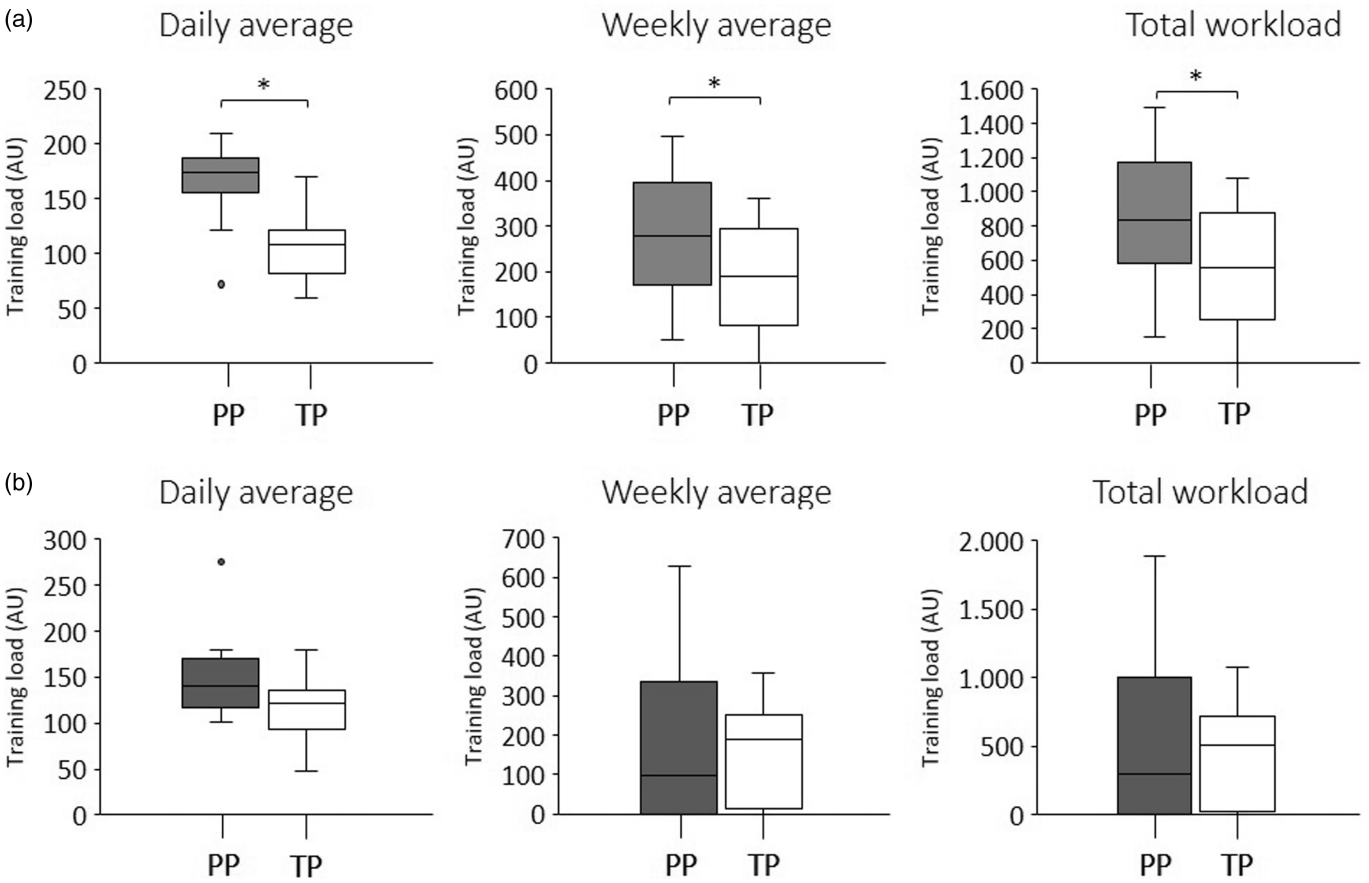
Workload comparison between synchronous and asynchronous tele-exercises. *Significant differences were found in the weekly and total workload comparisons (*p*≤0.05). AU: arbitrary units.

Average weekly and total workload were significantly higher in the synchronous compared to asynchronous tele-exercises (246.9 AU vs 153.3 AU and 740.5 AU vs 397.0 AU, respectively). Average daily workload did not present significant differences between the tele-exercises (Table 2 and Figure 4).

**Figure 5.**
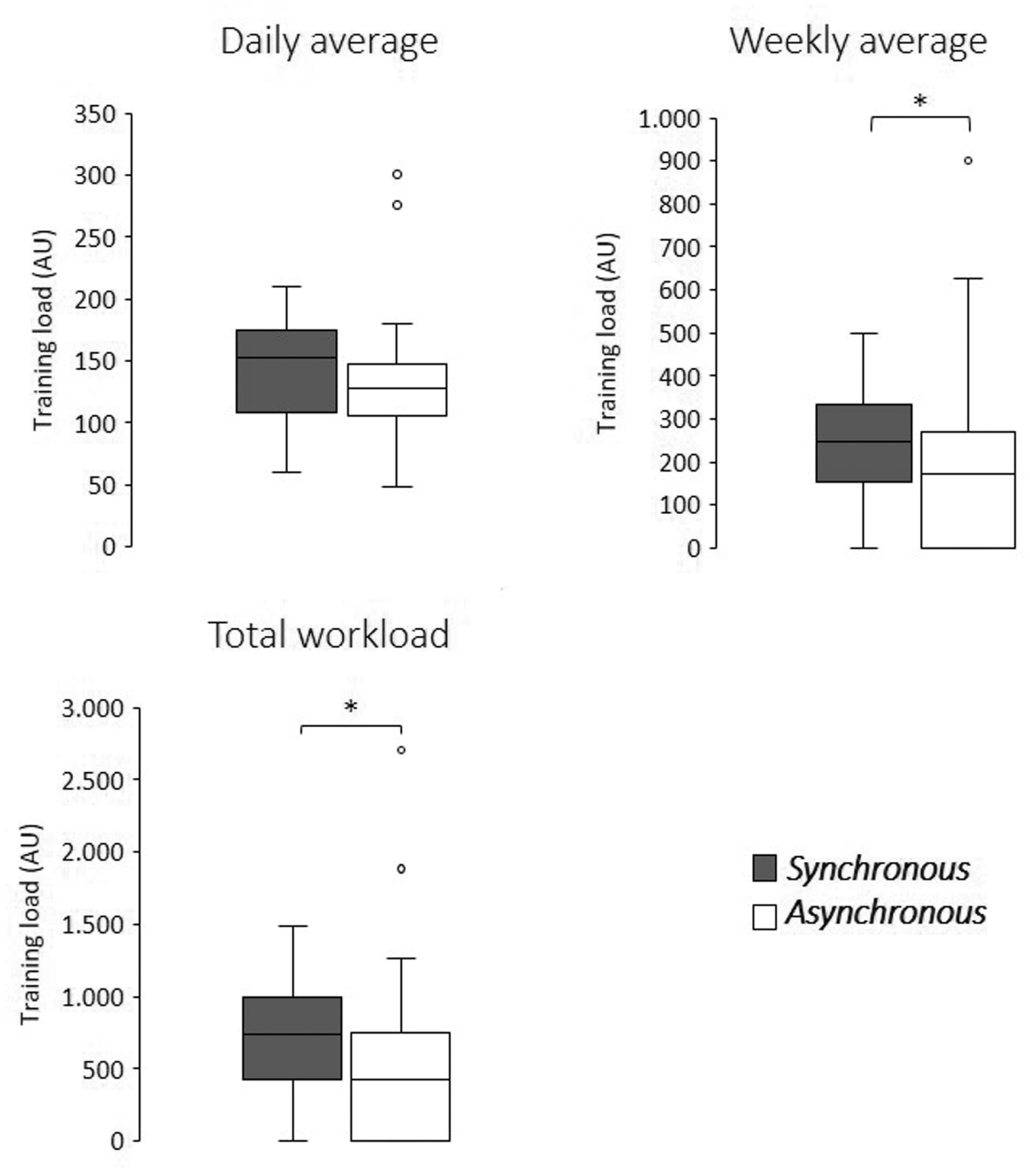
Workload comparison between paraplegic (grey boxplot) and tetraplegic (white boxplot) individuals in the synchronous tele-exercises (a) and asynchronous tele-exercises (b). Note that there were significant differences only in synchronous tele-exercise comparisons. *Significant differences were found in all workload variables in synchronous tele-exercises (*p* ≤ 0.05). AU: arbitrary units; PP: paraplegia; TP: tetraplegia.

In comparisons between paraplegia and tetraplegia groups, all training load variables in the synchronous format showed significantly higher values in the paraplegia group compared to the tetraplegia group. In asynchronous tele-exercise, there were no significant differences between paraplegia and tetraplegia groups for the training load variables (Table 2 and Figure 5).

## Discussion

The synchronous training presented more favourable implementation values, but the daily training load did not demonstrate any difference between the tele-exercises. In addition, the weekly and total values were higher in synchronous training since they were corrected by adherence, which was significantly higher in this form of tele-exercise. These results are important in view of the weekly training load ratios for beneficial effects on health promotion. The training load variables were higher in the paraplegia group, as expected, but there was no difference for implementation when compared with the tetraplegia group.

The implementation outcome was evaluated using two variables: adherence and successful data recording. Adherence showed significantly higher values in synchronous tele-exercise (66.7%) when compared to asynchronous tele-exercise (50.0%). In a previous study, Lai et al. included four participants and found 100% adherence, with 8% of the training sessions rescheduled.^16^ Compared to these data, the present study showed lower values; however, we included a larger sample (40 participants) and also the absence of rescheduling. Despite these differences, it can be inferred that synchronous tele-exercises, both individually and in groups, lead to greater adherence compared to asynchronous tele-exercises. Bombardier et al. also reported low exercise participation in a 16-session telehealth asynchronous programme.^30^

With respect to the successful recording of data, significantly higher values were found in the synchronous format (100.0%) than in the asynchronous format (71.4%). A previous study found values of 85%, with the reasons for the percentage difference being instability of Internet connection and mistaken recording of data.^16^ Although there was no systematic assessment of the reasons in the present study, the participants reported 'forgetting' as a factor for the lack of data recording. One of the aspects that can be highlighted is that individuals with tetraplegia could have greater difficulty in recording data due to the greater severity of SCI. However, the level of SCI did not interfere in the data recording, considering that there was no significant difference between the tetraplegia and paraplegia groups.

The daily training load did not show any significant difference between the synchronous and asynchronous tele-exercise formats. This finding offers consistency for the use of asynchronous tele-exercise, since this intervention format offers an opportunity for unstable Internet connection contexts in which exercise data can be saved after disconnecting from the Internet and resumed when the connection is restored.^16^ Asynchronous communication also enables the participant in the tele-exercise programme to remain guided by the professional even if they do not participate in activities in real time, avoiding the feeling of not being supervised.^36,37^ On the other hand, in terms of longitudinal monitoring, the asynchronous format may have limitations, since the weekly and total training load values were lower than in the synchronous tele-exercise. This is due to the impact on the training load associated with lower adherence to asynchronous tele-exercise. For example, to obtain cardiometabolic benefits related to exercise, it is estimated that a weekly training load of at least approximately 270–360 AU is necessary (30 min of moderate to vigorous activity, RPE 3–4, three times per week).^14^ In this context, weekly training loads closest to the desired levels were achieved in the synchronous training, although still below the level estimated for the population with SCI. Thus, despite the advantages described above regarding the use of the asynchronous approach, this method should be considered with caution in view of the limitations in chronic gains that can arise from lower adherence. Future studies evaluating different forms of monitoring asynchronous training may provide alternatives for an increase in the weekly and total training load in this form of tele-exercise.

Another finding refers to the absence of a significant difference in the training load between the paraplegia and tetraplegia groups in asynchronous tele-exercise, in addition to the higher values for the paraplegia group in the synchronous format. Individuals with paraplegia have greater muscle strength (greater preserved muscle mass), better body composition and less autonomic changes compared to individuals with tetraplegia.^31,38,39^ Dysautonomia is a most common issue in individuals with tetraplegia, compromising, for example, an increase in heart rate,^31,39^ and may consequently underestimate the values of RPE. Specific alterations according to the levels of injury can thus provide greater recruitment of muscle mass and cardiac demand and, as a consequence, higher values of RPE and training load compared to the tetraplegic group in synchronous tele-exercise. With respect to the absence of differences in training load in the asynchronous format, it can be inferred that paraplegics had greater difficulty in maintaining training intensity with the absence of the professional in real time. The relationship with the professional who conducts synchronous tele-exercise is considered a critical component for motivation in the activity,^16^ a fact which was more evident in the paraplegia group, to maintain higher values of RPE.

A differential aspect of this study was that it is the only one found in the literature that presents muscle strength training by synchronous tele-exercise collectively and not individually. Only studies on synchronous tele-exercise with SCI on an individual basis were found.^16,40^ For collective tele-exercise, a study with Tai Chi exercises was found with older adults^41^ and another using adapted dance in individuals with Parkinson's disease.^42^ Thus, the present study provides an alternative, using tele-exercise muscle strength training in groups for individuals with different levels of SCI. In addition, the intervention was shown to be safe, since no adverse effects were reported by the participants.

### Study limitations

Some characteristics of the sample must be considered before extrapolating conclusions regarding implementation. Participants had already taken part in a face-to-face rehabilitation programme and were young adult individuals, which may have favoured implementation due to the ease of accessibility to technological tools. Besides that, the present study performed a crossover without randomization that could have biased the results and, probably, this methodological approach might have increased the implementation data. Future studies could evaluate groups with different age groups and participants with follow-up in an exclusively remote rehabilitation programme associated with training and implementation loads.

## Conclusion

The training load for each training session did not differ between synchronous and asynchronous tele-exercises in individuals with SCI. Both adherence and successful data recording implementation values were more favourable in synchronous training, thus allowing greater weekly training loads (total and average). In this way, synchronous tele-exercise provides training load values that can more adequately correlate with beneficial effects to health promotion and with greater ease of implementation. One suggestion is to expand the forms of monitoring in asynchronous training in an attempt to promote greater adherence and, thus, higher weekly training loads. In addition, this analysis should be weighted by the level of injury, given that individuals with paraplegia achieved higher values in all outcomes related to training load compared to individuals with tetraplegia.
